# Life-Space Mobility and Objectively Measured Movement Behavior in Older Adults with Hypertension after Receiving COVID-19 Vaccination

**DOI:** 10.3390/ijerph191912532

**Published:** 2022-10-01

**Authors:** Rodrigo A. V. Browne, Ludmila L. P. Cabral, Gledson T. A. Oliveira, Geovani A. D. Macêdo, Júlio Sócrates, Raíssa de M. Silva, Maria B. F. Araújo, Yuri A. Freire, Eduardo C. Costa

**Affiliations:** 1 Graduate Program in Health Sciences, Federal University of Rio Grande do Norte, Natal 59012-570, Brazil; 2 Graduate Program in Physical Education, Federal University of Rio Grande do Norte, Natal 59078-970, Brazil; 3 Department of Physical Education, Federal University of Rio Grande do Norte, Natal 59078-970, Brazil

**Keywords:** coronavirus, social distancing, physical activity, sedentary behavior, life-space assessment

## Abstract

This study examined the changes in life-space (LS) mobility and objectively measured movement behavior in older adults with hypertension after receiving the COVID-19 vaccine and their associations with housing type. A total of 32 participants were included in this exploratory longitudinal study with a 1-year follow-up. LS mobility and accelerometer-based physical activity (PA) and sedentary behavior (SB) were assessed before and ~2 months after receiving COVID-19 vaccination. Participants residing in apartment/row housing showed an increase in LS mobility composite score (β = 14, *p* < 0.05). In addition, they showed an increase in light PA on weekdays and the weekend (β = 3.5%; β = 6.5%; *p* < 0.05) and a decrease in SB on weekdays and the weekend (β = −3.7%; β = −6.6%; *p* < 0.05). Furthermore, changes in SB pattern were found (less time spent in bouts of ≥10 and 30 min, more breaks/day and breaks/hour). Significant associations were found between changes in LS mobility composite score and PA (positive association) and SB (negative association) in older adults residing in apartment/row housing (*p* < 0.05). Older adults with hypertension, particularly those who resided in houses with limited outdoor space (apartment/row housing), showed positive changes in LS mobility and objectively measured movement behavior in a period after receiving the COVID-19 vaccine and characterized by social distancing policies without mobility restrictions when compared with the period of social distancing policies with high mobility restrictions and without vaccine.

## 1. Introduction

As of August 2022, the coronavirus disease 2019 (COVID-19) pandemic has already caused more than 6.4 million deaths worldwide [1]. Social distancing was the main public policy before the COVID-19 vaccine to mitigate virus transmission [2]. Social distancing policy is characterized by restricted mobility in public areas and the recommendation to “stay at home” [2]. Both of these recommendations were even more emphasized for groups at increased risk for severe COVID-19, such as older adults and individuals with chronic conditions [3,4]. Studies during this initial pandemic scenario have observed a reduction in life-space (LS) mobility in older adults [5,6,7], which is defined as the physical and social environment an individual inhabits and moves within on a day-to-day basis considering their frequency and independence [8]. In addition, we have observed unhealthy changes in objectively measured movement behavior in older adults with hypertension (i.e., less time spent on physical activity (PA) and more time spent on sedentary behavior (SB)) [9]. Several systematic reviews have confirmed these findings [10,11,12]. Interestingly, the unhealthiest changes in movement behavior occurred on the weekend and particularly in individuals who resided in apartments and row housing (characterized by limited outdoor space) when compared with those who resided in detached houses [13]. Of note, all studies that reported a reduction in LS mobility [5,6,7] and unhealthy changes in objectively measured movement behavior in older adults [9,13] were conducted in a period prior to the COVID-19 vaccines.

The COVID-19 vaccines are safe and have a high efficacy rate in clinical trials [14,15,16,17] and real-world settings [18,19,20,21,22,23,24,25,26] against serious COVID-19-related illnesses, hospitalization, and death. Massive immunization has allowed individuals to gradually return to their activities outside the home environment [27]. However, it is still unclear whether this new stage in the COVID-19 pandemic has had an impact on LS mobility and objectively measured movement behavior, especially in older adults. Therefore, this exploratory longitudinal study investigated the changes in LS mobility and objectively measured movement behavior in older adults with hypertension after COVID-19 vaccination and their associations with housing type. We hypothesized that an increase in LS mobility and healthy changes in objectively measured movement behavior (more PA, less SB) would occur after COVID-19 vaccination, particularly in older adults who resided in housing with limited outdoor space (apartment/row housing).

## 2. Materials and Methods

### 2.1. Study Design

This is an exploratory longitudinal study that is a 1-year follow-up of a previous study that examined the initial impact of the COVID-19 pandemic on objectively measured movement behavior in older adults with hypertension involved in an interrupted clinical trial [9,13]. The current study was designed to investigate the LS mobility and objectively measured movement behavior before and after the COVID-19 vaccine in a group of older adults with hypertension and their associations with housing type. The study was carried out in the city of Natal, which has ~900 thousand habitants and is the capital city of Rio Grande do Norte, one of the 9 northeast states of Brazil. The baseline measurements occurred in June 2020, and the 1-year follow-up measurements occurred in July 2021. The weather climate was similar between the two collection periods.

### 2.2. Participants

The sample consisted of Brazilian older adults with hypertension who had participated in two previous studies [9,13]. A total of 35 participants were invited to participate in the study, but 3 participants declined to participate (one 1 personal reasons and 2 who were traveling at the time of data collection). Thus, 32 participants were included in the final analysis. These participants were screened for a clinical trial that was interrupted due to the COVID-19 pandemic (for more details, see Browne et al. [9]). The eligibility criteria for the interrupted clinical trial are available in the Brazilian Clinical Trials Registry (registration number: RBR-4ntszb). For more details, see: http://ensaiosclinicos.gov.br/rg/RBR-4ntszb/ (accessed on 29 July 2022). The main eligibility criteria were: aged 60–80 years; medical diagnosis of hypertension; not being engaged in a regular PA program; being physically inactive; absence of previous cardiovascular events or other cardiovascular diseases; absence of uncontrolled hypertension; absence of contraindications to exercise; and absence of neurological progressive disorders. Most of these participants met the eligibility criteria of the clinical trial, except eight participants who were considered physically active by accelerometer measurement. However, the eight physically active participants were included for the current exploratory longitudinal study.

### 2.3. Epidemiological Scenario before and after COVID-19 Vaccination

The baseline measurements occurred on June 2020, a period of high mobility restriction in the public areas, which included a public recommendation to stay at home, especially for older adults and high-risk individuals [9]. In addition, none of the participants had received the COVID-19 vaccine. On 1 June 2020, Brazil had 526,447 and 29,937 COVID-19-related confirmed cases and deaths, respectively [28]. In addition, the numbers of COVID-19-related confirmed cases and deaths grew steadily [29]. In the city of Natal, there were 3103 and 105 COVID-19-related confirmed cases and deaths, respectively [28]. The number of cases increased [30], and the moving average occupancy rate of intensive care unit (ICU) beds was 91.7% [31]. The 1-year follow-up measurement occurred on 7 July 2021, after the participants had taken at least one dose of COVID-19 vaccine and amid less severe social distancing policies, at which time there were 18,909,037 and 528,540 COVID-19-related confirmed cases and deaths in Brazil, respectively [28]. The numbers of COVID-19-related confirmed cases and deaths was decreasing [32]. There were 95,155 and 2546 COVID-19-related confirmed cases and deaths in the city of Natal, respectively [28]. The number of cases decreased [33], and the moving average rate of ICU beds was 57.1% [31].

### 2.4. Study Procedures

The baseline assessments were performed in June 2020, including: (i) a questionnaire about medical history, medication, and sociodemographic and behavioral characteristics; among the behavioral questions, the following questions were asked: Did you leave home? If yes, where did you go and how often? (ii) a questionnaire about the housing characteristics; and (iii) accelerometer-based PA and SB measures. In addition, characteristics related to COVID-19 were registered over the course of the study follow-up, including cases of infection, symptoms, vaccination, and type of vaccine. The follow-up assessments were performed 1 year later in July 2021, including: (i) accelerometer-based PA and SB measures; (ii) a questionnaire to measure LS mobility referring to the month of June 2020 (recall); before applying this questionnaire, the behavioral responses from June 2020 were remembered; (iii) a questionnaire to measure LS mobility referring to the month of July 2021.

### 2.5. Housing Characteristics

Information about housing characteristics that the participants were residing in during the COVID-19 pandemic, including housing type, housing surface area (m^2^), and household size (i.e., number of persons residing in the home) were collected by phone in June 2020. Housing type was categorized into apartment, row house, or detached house [34,35,36,37]. In the current study, the small subgroup of participants who resided in an apartment (*n* = 5) was combined with the subgroup of participants who resided in a row house (*n* = 10), since both types are characterized by limited outdoor areas, as well as have similar housing surface areas (*p* > 0.05). Furthermore, a previous study by our research group demonstrated that participants who resided in apartments and row houses had greater and similar unhealthy changes in objectively measured movement behavior than did participants who resided in a detached house during the COVID-19 pandemic [13]. Housing surface area was categorized by tertiles: ≤105 m^2^, 106–249 m^2^, and ≥250 m^2^. Household size was categorized into one or two persons or three or more persons.

### 2.6. Objectively Measured Movement Behavior

All participants who agreed to participate in this study received sterilized accelerometers in their homes to be used over 7 consecutive days. Movement behavior measures, including PA and SB, were assessed by accelerometer (GT3X, Actigraph LLC, Pensacola, FL, USA). All participants were instructed to wear the accelerometer on their right hip during 7 consecutive days, including awake and asleep periods, and to remove it during bathing. The position of the accelerometer on the participant’s body was verified through a photo or videoconference. They also filled out a diary describing the time they took off the accelerometer during the awake period, when they went to bed, and when they woke up. A sampling rate of 60 Hz with a period of 60 s was used. Non-wearing time was defined as ≥90 consecutive minutes of 0 counts with a tolerance of up to 2 min of ≥100 counts/min [38]. Participants with at least 3 valid weekdays of accelerometer wearing time (≥600 min/day) with at least 1 weekend day, totaling at least 4 valid days, were included in the data analysis [39]. Despite these strict guidelines, all participants had 4–5 valid days of accelerometer use on weekdays and 2 valid days on weekend in both data collection periods. Although there is no consensus on the cut-off points for older adults, we used those that are most reported in this population [40]. The cut-offs in counts per minutes to define SB, light PA, and moderate-vigorous PA were: 0–99, 100–1951, and ≥1952, respectively [41,42]. Accelerometer-based PA and SB measures were analyzed as weekdays and weekend using the ActiLife version 6.13.3.2 software program. The following variables were considered for data analysis: steps/day; time spent in SB, light PA, and moderate-vigorous PA (min/day and accelerometer wear time %); bouts ≥ 10 and 30 consecutive minutes of SB, light PA, and moderate-vigorous PA (min/day and bouts/day); length of sedentary bouts (min/day; defined as time spent in SB ÷ number of sedentary bouts ≥ 1 min); sedentary breaks of ≥1 and ≥5 min (breaks/day; defined as ≥100 counts/min following a sedentary bout); break rate (breaks/hour; defined as number of breaks ÷ accelerometer wear time) [43].

### 2.7. Life-Space Mobility

The Brazilian version of the LS Assessment Questionnaire [44] was used to measure LS mobility by phone. The questionnaire contains five LS levels: level 1—outside the bedroom; level 2—outside the home; level 3—neighborhood; level 4—city; level 5—other cities. The participants were assessed for displacement in the previous 4 weeks. Participants were asked how often each level was reached, as well as whether it was achieved independently. A LS composite score was calculated multiplying each level (1 to 5) achieved by frequency (i.e., 1 = <1 time/week; 2 = 1–3 times/week; 3 = 4–6 times/week; and 4 = daily) and independence level (i.e., 1 = personal assistance, 1.5 = assistive devices, 2 = no assistance). The score ranges from 0 to 120, and higher scores indicate greater mobility [44,45]. In addition to the composite score, a maximum LS score was calculated (range 0 to 5) that measures the highest level of living space achieved regardless of the assistance (i.e., assistive devices or personal assistance). Participants were stratified in relation to LS mobility restriction, defined as a LS composite score ≤ 60 points [46]. Participants were also stratified in relation to the increase in LS composite score, for which a 10-point increase was considered clinically important [47,48,49].

### 2.8. Statistical Analysis

Descriptive data of the characteristics of the participants are presented as mean ± standard deviation or absolute and relative frequencies. A generalized gamma model with robust variance and Fisher’s exact test were used to compare the participants’ characteristics between the housing type groups. A generalized linear mixed model with the subject as a random effect and the housing type, time period, and covariates as fixed effects was used to assess the housing types associated with changes in objectively measured movement behavior measures, controlling for the following covariates: (a) only by accelerometer wear time and then by (b) age, gender, education, income, employment status, and accelerometer wear time. The subject was included as a random effect due to the high intra-subject variability in the objectively measured movement behavior measures. A generalized linear model with housing type, time period, and covariates as fixed effects was used to assess the housing types associated with changes in LS mobility measures controlling for age, gender, education, income and employment status. A generalized linear mixed model with subject as a random effect and increased LS (binary variable; i.e., 10-point increase in LS composite score) and time period as fixed effects was used to assess the increased LS associated with changes in objectively measured movement behavior scores in the group of participants residing in an apartment/row house. Robust estimation was used for the fixed-effects model. The model results were expressed as estimated marginal means (EMM), coefficient or contrast estimates (β), and 95% Wald confidence interval (CI). The residuals’ distribution was verified using the normal Q-Q plot. A Poisson regression model with robust variance was used to assess changes in the prevalence of restricted LS. The model results were expressed as prevalence ratio (PR) and 95% Wald CI. A two-tailed *p* < 0.05 was considered statistically significant for all analyses. Statistical analyses were performed using IBM SPSS Statistics for Win/v.27.0 (IBM Corp., Armonk, NY, USA).

## 3. Results

### 3.1. Participant Characteristics

Table 1 shows the characteristics of the included participants. Most of the participants were women (66%, *n* = 21), lived with a partner (50%, *n* = 16), were retired (69%, *n* = 22) and considered their income suitable/very good (63%, *n* = 20), and resided in a detached house (53%, *n* = 17). Approximately 47% (*n* = 15) of the participants resided in a non-detached house, i.e., an apartment (16%, *n* = 5) or row house (31%, *n* = 10). All participants were taking anti-hypertensive medication(s). Approximately 41% (*n* = 13) of the participants had type 2 diabetes, and 41% (*n* = 13) had dyslipidemia. Most participants were physically inactive (78%, *n* = 25; <150 min/week of moderate-vigorous PA). A total of 31% (*n* = 10) had had COVID-19 between May/2020 and May/2021, which was before the second data collection (July 2021). None of the participants infected with COVID-19 required hospitalization or had severe symptoms. None of the participants was infected or they were in post-COVID-19 recovery at the second data collection. All participants had received at least one dose of the COVID-19 vaccine before the second data collection, between February and May 2021 (i.e., 34% (*n* = 11) were partially vaccinated and 66% (*n* = 21) were fully vaccinated). No significant differences were found between participants who resided in apartment/row housing and those who resided in a detached house, except for the housing surface area: The detached houses had a larger housing surface area than the apartment/row housing (β = 169 m^2^, 95% CI 111, 228, *p* < 0.001).

### 3.2. Changes in Life-Space Mobility after COVID-19 Vaccination

Regarding the associations between housing type and changes in LS mobility scores after the COVID-19 vaccine, there was no moderating effect of housing type on changes in LS mobility scores (*p* < 0.05). However, the data analyses were conducted according to the housing type due to the significant moderating effect of housing type on changes in PA and SB volume and pattern (see Section 3.3 for more details).

**Figure 1:**
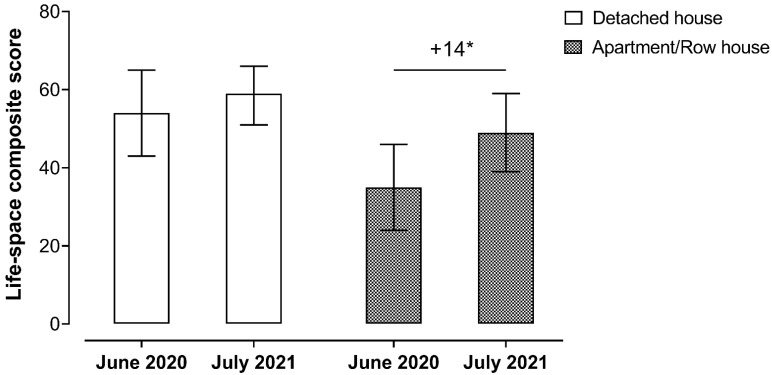
Life-space mobility composite score before and after COVID-19 vaccination among 32 older adults with hypertension residing in a detached house (*n* = 17) or apartment/row housing (*n* = 15). Values are expressed as mean and its 95% Wald confidence interval. The models were analyzed using a generalized linear model. * *p* < 0.05.

Concerning the group of participants residing in apartment/row housing, there was a significant increase in LS composite score after COVID-19 vaccination (35 (95% CI 24, 46) vs. 49 (95% CI 39, 59); β = 14, 95% CI 3, 24, *p* = 0.011) (Figure 1). There was also a significant increase in maximum LS after COVID-19 vaccination (2.9 (95% CI 2.4, 3.4) vs. 3.6 (95% CI 3.2, 4.0); β = 0.7, 95% CI 0.3, 1.2, *p* = 0.002). No significant change was observed for the prevalence of restricted LS (score ≤ 60) after COVID-19 vaccination (80.0% (*n* = 12) vs. 66.7% (*n* = 10); PR = 1.2, 95% CI 0.8, 1.9, *p* = 0.415). No significant change in the group of participants residing in a detached house was observed for the LS composite score (54 (95% CI 43, 65) vs. 59 (95% CI 51, 66); β = 5, 95% CI −3, 13, *p* = 0.237) and maximum LS (3.8 (95% CI 3.3, 4.3) vs. 4.2 (95% CI 4.0, 4.5); β = 0.4, 95% CI −0.1, 0.9, *p* = 0.086). In addition, there was no increase in prevalence of restricted LS (score ≤ 60) for the detached house (58.8% (*n* = 10) vs. 47.1% (*n* = 8); PR = 1.3, 95% CI 0.7, 2.4, *p* = 0.496). Finally, the changes in LS mobility scores were tested according to the housing type controlling for age, gender, education, income, and employment status. However, the results remained unchanged after including these covariates.

### 3.3. Moderating the Effect of Housing Type on Objectively Measured Changes in Movement Behavior after COVID-19 Vaccination

The moderating effect of housing type on changes in the volume of PA and SB after the COVID-19 vaccine (i.e., change in apartment/row housing vs. change in detached house—reference group) is shown in Table S1. There was a trend towards significance regarding the weekdays for a greater increase in light PA and a greater decrease in SB in participants who resided in apartment/row housing compared with those resided in a detached house (*p* < 0.10). Participants who resided in apartment/row housing showed a greater increase in light PA (*p* < 0.05) on the weekend and a trend towards significance for a greater decrease in SB compared with those who resided in a detached house (*p* < 0.10). No moderating effect of housing type was found on the changes in moderate-vigorous PA or steps/day on weekdays and the weekend.

The moderating effect of housing type on changes in PA and SB patterns after COVID-19 vaccination is shown in Table S2. There was a trend towards significance on weekdays for greater decreases in sedentary bouts ≥ 30 min, length of sedentary bouts, and number of breaks and break rate ≥ 5 min from SB in participants who resided in apartment/row housing compared with those resided in a detached house (*p* < 0.10). Participants who resided in apartment/row housing showed a greater decrease in sedentary bouts ≥ 10 and 30 min and length of sedentary bouts and a greater increase in number of breaks and break rate ≥ 5 min from SB on the weekend (*p* < 0.05).

### 3.4. Objectively Measured Changes in the Volume of Movement Behavior after COVID-19 Vaccination

**Figure 2:**
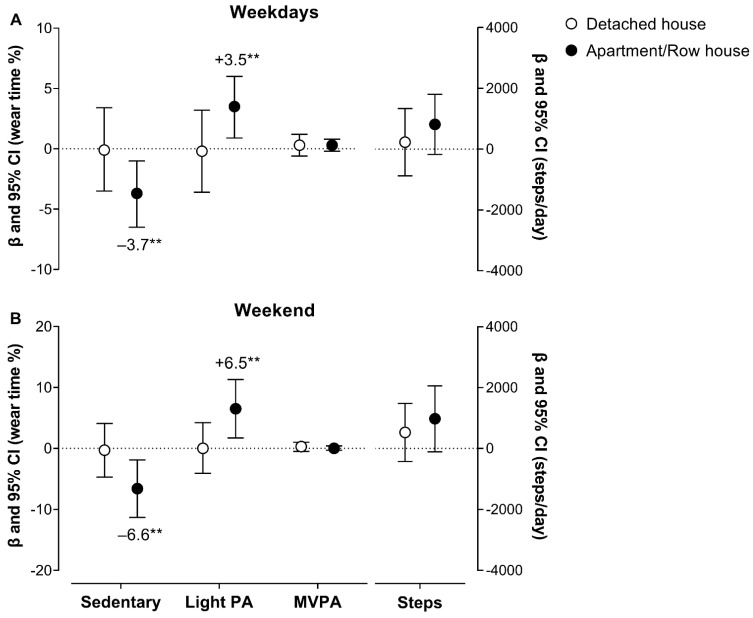
Objectively measured changes in the physical activity and sedentary behavior volume on weekdays (panel **A**) and the weekend (panel **B**) after COVID-19 vaccination among 32 older adults with hypertension residing in a detached house (*n* = 17) or an apartment/row house (*n* = 15). Values are expressed as contrast estimates (β) and the 95% Wald confidence interval (CI) of the estimated marginal mean (EMM) (contrast = EMM of after—EMM of before the COVID-19 vaccine). The models were analyzed using a generalized linear mixed model (steps per day models were controlled for the daily accelerometer wear time). ** *p* < 0.01. Abbreviations: MVPA, moderate-vigorous physical activity; PA, physical activity.

Figure 2 and Table 2 shows the changes in the PA and SB volume after COVID-19 vaccination according to the housing type. Concerning the changes in the group of participants residing in an apartment/row house, there was a significant decrease in SB (β = −3.7%, *p* = 0.008) and an increase in light PA (β = 3.5%, *p* = 0.009) on weekdays. These changes showed a strong correlation (*r* = −0.98, *p* < 0.001). No significant changes were found for moderate-vigorous PA or steps/day on weekdays (*p* > 0.05). There was a significant decrease in SB (β = −6.6%, *p* = 0.007) and an increase in light PA (β = 6.5%, *p* = 0.008) on the weekend. These changes also showed a strong correlation (*r* = −0.99, *p* < 0.001). There was a trend towards an increase in steps/day (β = 976, *p* = 0.077). No significant changes were found for the group of participants residing in a detached house on weekdays or the weekend (*p* > 0.05). In addition, changes in the PA and SB volume and pattern were tested according to the housing type controlling for age, gender, education, income, employment status, and daily accelerometer wearing time. However, the results remained unchanged after including these covariates.

### 3.5. Objectively Measured Changes in the Movement Behavior Pattern after COVID-19 Vaccination

**Figure 3:**
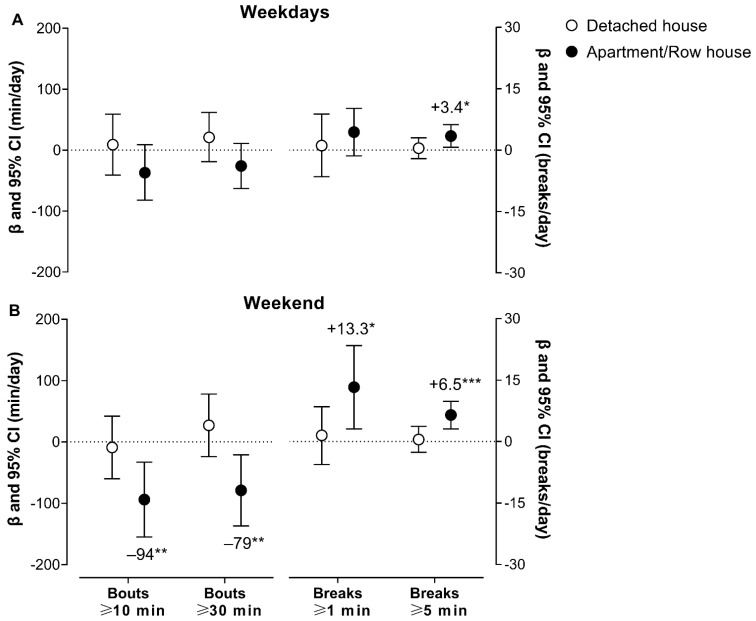
Changes in sedentary bouts and breaks on weekdays (panel **A**) and on the weekend (panel **B**) after COVID-19 vaccination among 32 older adults with hypertension residing in a detached house (*n* = 17) or apartment/row housing (*n* = 15). Values are expressed as contrast estimates (β) and the 95% Wald confidence interval (CI) of the estimated marginal mean (EMM) (contrast = EMM of after—EMM of before COVID-19 vaccination). The models were analyzed using a generalized linear mixed model controlling for the daily accelerometer wearing time. * *p* < 0.05; ** *p* < 0.01; *** *p* < 0.001.

Figure 3 and Table 3 shows the changes in the PA and SB pattern after COVID-19 vaccination according to the housing type. Concerning the changes in the group of participants residing in apartment/row housing, there were increases in the number of breaks ≥ 5 min from SB (β = 3.5 breaks/day, *p* = 0.015) and the break rate on the weekdays. There were significant decreases in sedentary bouts ≥ 10 and 30 min (β = −94 min/day; β = −79 min/day; *p* < 0.05) and the length of sedentary bouts on the weekend. There were also increases in the number of breaks ≥ 1 and 5 min from SB (β = 13.3 breaks/day; β = 6.5 breaks/day; *p* < 0.05) and the break rate. No significant changes were found for the group of participants residing in a detached house on weekdays or the weekend (*p* > 0.05). In addition, the changes in the PA and SB patterns were tested according to the housing type controlling for age, gender, education, income, employment status, and daily accelerometer wearing time. However, the results remained unchanged after including these covariates.

### 3.6. Associations of Changes in Life-Space Mobility with Objectively Measured Changes in Movement Behavior

There were significant associations of increased LS mobility (binary variable; i.e., 10-point increase of LS composite score) with the changes in PA and SB outcomes on weekdays and the weekend after the COVID-19 vaccine in older adults with hypertension residing in apartment/row housing (Tables S3 and S4). Table S3 shows the associations of increased LS mobility with the changes in the PA and SB volumes. Regarding weekdays, there was a significant association of increased LS with an increase in steps/day (*p* < 0.05) and a trend towards significance for an increase in moderate-vigorous PA (*p* < 0.10). There was a trend towards significance for the association of increased LS with an increase in light PA and a decrease in SB on the weekend (*p* < 0.10). Table S4 shows the associations of increased LS mobility with the changes in the PA and SB patterns. Moreover, no associations of increased LS with changes in the PA and SB patterns were observed for weekdays. However, there was a significant association of increased LS with a decrease in bouts ≥ 10 min for SB and increases in breaks and break rate ≥ 5 min on the weekend (*p* < 0.05). In addition, there was a significant association of increased LS with an increase in bouts ≥ 10 min for light PA (*p* < 0.05). No significant associations of change in LS mobility composite score with PA and SB outcomes on weekdays and the weekend were observed (*p* > 0.05).

The exploratory analysis of associations of increased LS mobility with changes in PA and SB after the COVID-19 vaccine were not performed in the older adults residing in a detached house since no significant change was found in the LS mobility or in the volume and pattern of PA and SB outcomes (*p* > 0.05).

## 4. Discussion

To the best of our knowledge, this exploratory longitudinal study is the first to report the changes in LS mobility and objectively measured movement behavior after receiving the COVID-19 vaccine in older adults with hypertension. The main findings indicate an increase in LS mobility and healthy changes in movement behavior in older adults with hypertension who resided in apartment/row housing after receiving the COVID-19 vaccine. In summary, the following were observed: (i) an increase in the LS mobility composite and maximum LS mobility scores; (ii) an increase in light PA and a decrease in SB on weekdays and weekend; (iii) a decrease in prolonged/uninterrupted SB and an increase in breaks of SB. Moreover, the increase in LS mobility was associated with an increase in PA and a decrease in SB.

The increase in maximum LS mobility among older adults residing in apartment/row housing after COVID-19 vaccination occurred in particular outside the home environment (i.e., ≥level 3—neighborhood). During the social distancing policy (June 2020) period, 80% of these older adults showed restricted LS mobility (score ≤ 60). This finding indicates that most of these older adults complied with the recommendation to stay at home. The social distancing policies imposed to mitigate the COVID-19 pandemic caused a reduction in the LS mobility of older adults [5,6,7]. However, COVID-19 vaccination may have provided greater security for the individuals to return to their regular activities, which were partially interrupted due to the COVID-19 pandemic, e.g., walking in shopping malls, squares, and parks; going to the supermarket; visiting friends and family; playing with grandchildren; and travelling. Indeed, the vaccination of older adults reduces the number of infections, hospitalizations, and deaths [14,15,16,17], which in turn promotes more security for moving outside the home. It seems reasonable that the perception of risk of being infected by coronavirus is reduced when vaccinated [50,51], which may partially explain the increase in LS mobility. In addition, older adults residing in apartment/row housing showed a clinically important increase in LS composite score (i.e., +14 points) after their COVID-19 vaccination, which takes into account the frequency and independence of mobility at each level achieved. Previous studies have shown that an increase of at least 10 points in the LS mobility composite score was associated with an increase in the performance of daily living activities and a reduction in the risk of health-related conditions, falls, hospital admissions, and death [6,48,52,53]. Taken together, the COVID-19 vaccine seems to have contributed to an increase in the LS mobility in older adults with hypertension.

Moreover, our findings demonstrate that the COVID-19 vaccination was associated with healthy changes in movement behavior, particularly in the older adults residing in apartment/row housing. As our previous study demonstrated [13], older adults who resided in apartment/row housing decreased PA more and increased SB more during the social distancing policy imposed due to the COVID-19 pandemic. On the other hand, the current study shows that only these older adults, and not those who resided in a detached house, increased PA and decreased SB after the COVID-19 vaccination. Our data suggest that the older adults who resided in apartment/row housing replaced ~30 and ~60 min of SB for light PA on weekdays and the weekend after COVID-19 vaccination, respectively. These changes in the time spent in light PA and in SB did not occur in the older adults resided in detached housing after receiving the COVID-19 vaccine, probably because during the period of social distancing policy, this group also did not show significant changes in these variables [13]. Interestingly, with the healthy changes in movement behavior after COVID-19 vaccination, the time spent in light PA (~280 min/day) and SB (~680 min/day) was similar between the older adults who resided in apartment/row housing and a detached house.

Although we did not find an increase in moderate-vigorous PA levels after COVID-19 vaccination, the changes found in light PA and SB in the older adults residing in apartment/row housing are clinically significant and seem to reflect a replacement of SB with light PA. Isotemporal substitution theoretical models shed light on the clinical importance of replacing SB with light PA, which occurred after COVID-19 vaccination in older adults with hypertension who resided in apartment/row housing. For example, in a cross-sectional study involving 2189 older adults with metabolic syndrome, the replacement of 30 min/day of SB with light PA was associated with lower BMI, waist circumference, total body fat, visceral adipose tissue, glycated hemoglobin, glucose and triglycerides and higher total body muscle mass and HDL-cholesterol [54]. A systematic review conducted by del Pozo-Cruz et al. [55] also indicated that replacing 30 min/day of SB with light PA was associated with 13 to 20% reductions in the risk of all-cause mortality. Furthermore, the reduction in prolonged/uninterrupted SB found in older adults residing in apartment/row housing after COVID-19 vaccination has the potential to improve cardiometabolic risk [56,57]. Thus, it is possible that the healthy changes in movement behavior observed after the COVID-19 vaccine may have positive impacts on cardiometabolic health in older adults residing in houses with limited outdoor area.

This study has strengths and limitations that should be mentioned. As strengths, the movement behavior was objectively measured using a triaxial accelerometer, which avoids the bias (underestimating or overestimating) of self-reporting PA and SB. In addition, the accelerometer enables measuring the PA and SB pattern, which is not possible using questionnaires. The longitudinal design enabled following the same participants over two different scenarios: before and after COVID-19 vaccination. As limitations, this is an exploratory longitudinal study that included participants who were originally screened for a clinical trial that was interrupted due to the COVID-19 pandemic. The interrupted clinical trial was not originally designed to examine the changes in LS mobility and objectively measured movement behavior during the COVID-19 pandemic. These limitations also made it impossible to recruit a larger sample. Although the housing types included in this study were similar to those used in other countries [34,35,36,37], we recognize that urban and sociocultural characteristics can make housing types incomparable among countries. Despite the sensitivity of the accelerometer settings to identifying the volume and pattern of SB, this equipment does not identify the participant’s posture; therefore, standing behaviors may be misclassified as SB. The LS mobility of June 2020 (baseline) was assessed by phone in 2021, and there may have been some recall error, although the participants did not report difficulty answering the questions related to their LS mobility 1 year ago. In addition to the COVID-19 vaccination, the epidemiological scenario of the pandemic was different between June 2020 and July 2021. During the second phase of the data collection (July 2021), the numbers of COVID-19-related confirmed cases, hospitalizations and deaths reduced, and therefore, the potential fear of COVID-19 may also have lessened (contributing to a greater sense of security) among older adults, leading to changes in their movement behaviors. Moreover, in this period, social distancing policies were less severe, allowing for the opening of shopping malls, squares and parks, and supermarkets among others, which could have stimulated changes in their activity behaviors. Therefore, this aspect per se could have contributed to the positive changes in the LS mobility and movement behavior observed in the current study. Finally, psychological aspects related to adherence to social distancing policies were not evaluated, so it is unknown whether after a period after the restrictions, participants stopped adhering to these policies, leading to more movement outside their home. Taken together, our preliminary results should be interpreted with caution.

### Practical Applications and Future Directions

Based on our findings, which indicate that in the scenario after COVID-19 vaccination and less severe social distancing policies, older adults with hypertension show healthy changes in movement behavior, we propose that professionals develop interventions that encourage a reduction in the time spent in SB and an increase in the time spent in PA regardless of its intensity, especially for those who reside in apartments/row housing, who were more vulnerable to unhealthy changes in movement behavior during the pandemic. Previously, during the period of social distancing policies with high mobility restrictions and without vaccination, we recommended several household countermeasures [9,13]. However, the current scenario in most countries and regions allows these interventions to be directed to an increase in total PA in different environments (e.g., community, square, beach) and contexts (e.g., leisure, dances, sports, active transportation), always taking the necessary sanitary precautions against contamination with COVID-19.

Even though our findings provide an understanding of changes in movement behavior and LS mobility across different scenarios of the COVID-19 pandemic among older adults with hypertension, we recommend future research directions for this topic. First, we emphasize the importance of future studies with a similar design (i.e., longitudinal study using objectively measured movement behavior) to replicate our findings with a larger sample that includes people of different cultures and education and socioeconomic levels, as well as different levels of PA and physical fitness and different body compositions. Second, researchers should consider including assessments of physical, psychological, and mental health outcomes to strengthen the understanding of the health impacts of movement behavior among older adults with hypertension.

## 5. Conclusions

Older adults with hypertension, particularly those who reside in houses with limited outdoor space (apartment/row housing), showed positive changes in LS mobility and objectively measured movement behavior in a period after receiving the COVID-19 vaccine that was characterized by social distancing policies without mobility restrictions when compared with a period of social distancing policies with high mobility restrictions and without a vaccine.

## Figures and Tables

**Table 1:** Characteristics of the participants according to the housing type.

	Detached House	Apartment/ Row House	*p* ^a^	Overall
*n* (%)	17 (53.1)	15 (46.9)		32
Age, yrs	67.5 ± 4.5	66.5 ± 3.5	0.467	67.0 ± 4.0
Female, (%)	9 (52.9)	12 (80.0)	0.147	21 (65.6)
Living with a partner, (%)	9 (52.9)	7 (46.7)	1.000	16 (50.0)
Living with grandchildren, (%)	5 (29.4)	2 (13.3)	0.402	7 (21.9)
Post-secondary education, (%)	4 (23.5)	3 (20.0)	1.000	7 (21.9)
Employment status, (%)				
Employed (home office)	4 (23.5)	6 (40.0)	0.450	10 (31.3)
Retired	13 (76.5)	9 (60.0)		22 (68.8)
Per capita income, (%)				
<1 MWS	4 (23.5)	4 (26.7)	0.906	8 (25.0)
1–2 MWS	7 (41.2)	7 (46.7)		14 (43.8)
>2 MWS	6 (35.3)	4 (26.7)		10 (31.3)
Income sufficiency (suitable/very good), (%)	12 (70.6)	8 (53.3)	0.467	20 (62.5)
Ex-smoker, (%)	6 (35.3)	2 (13.3)	0.229	8 (25.0)
Physically inactive, (%)	12 (70.6)	13 (86.7)	0.402	25 (78.1)
Type 2 diabetes, (%)	9 (52.9)	4 (26.7)	0.166	13 (40.6)
Dyslipidemia, (%)	7 (41.2)	6 (40.0)	1.000	13 (40.6)
Hypertension diagnosis, yrs	15.4 ± 11.9	10.8 ± 4.0	0.121	13.3 ± 9.3
COVID-19 infected, (%)	6 (35.3)	4 (26.7)	0.712	10 (31.3)
COVID-19 vaccine, (%)				
CoronaVac	12 (70.6)	9 (60.0)	0.450	21 (65.6)
AstraZeneca/Oxford	4 (23.5)	6 (40.0)		10 (31.3)
Pfizer	1 (5.9)	0 (0.0)		1 (3.1)
Partially vaccinated, (%)	6 (35.3)	5 (33.3)	1.000	11 (34.4)
Fully vaccinated, (%)	11 (64.7)	10 (66.7)		21 (65.6)
Household size, (%)				
1–2 persons	11 (64.7)	10 (66.7)	1.000	21 (65.6)
3+ persons	6 (35.3)	5 (33.3)		11 (34.4)
Housing surface area, m^2^	284 ± 103	115 ± 69	**<0.001**	205 ± 123
Housing surface area, (%)				
≤105 m^2^	1 (5.9)	10 (66.7)	**<0.001**	11 (34.4)
106–249 m^2^	7 (41.2)	5 (33.3)		12 (37.5)
≥250 m^2^	9 (52.9)	0 (0.0)		9 (28.1)

Values expressed as mean ± standard deviation or absolute and relative (%) frequencies. ^a^ Generalized gamma model and Fisher’s exact test were used for the analysis of continuous and categorical data, respectively. Bold values indicate significance at *p* < 0.05. Abbreviations: COVID-19, Coronavirus Disease 2019; MWS, minimum wage salary.

**Table 2:** Volumes of objectively measured physical activity and sedentary behavior before and after COVID-19 vaccination in older adults with hypertension according to the housing type.

	Detached House				Apartment/Row House			
	June 2020	July 2021			June 2020	July 2021		
	EMM (95% CI)	EMM (95% CI)	β (95% CI) ^a^	*p*	EMM (95% CI)	EMM (95% CI)	β (95% CI) ^a^	*p*
**SEDENTARY BEHAVIOR**								
**Weekdays**								
Sedentary, wear time %	69.5 (65.5, 73.5)	69.5 (66.6, 72.3)	−0.1 (−3.5, 3.4)	0.970	73.9 (70.1, 77.6)	70.1 (66.3, 74)	−3.7 (−6.5, −1)	**0.008**
Sedentary, min/day	687 (648, 727)	688 (660, 716)	1 (−36, 38)	0.963	728 (693, 764)	698 (659, 737)	−31 (−62, 0)	0.052
**Weekend**								
Sedentary, wear time %	70.2 (65.5, 74.8)	69.8 (65.4, 74.3)	−0.3 (−4.7, 4.1)	0.890	74.9 (69.5, 80.3)	68.3 (64.9, 71.7)	−6.6 (−11.3, −1.9)	**0.007**
Sedentary, min/day	660 (618, 702)	660 (616, 704)	−1 (−44, 43)	0.976	706 (657, 755)	645 (614, 676)	−61 (−104, −17)	**0.007**
**PHYSICAL ACTIVITY**								
**Weekdays**								
Light PA, wear time %	28.8 (25.5, 32.1)	28.6 (25.6, 31.5)	−0.2 (−3.6, 3.2)	0.903	25.1 (21.3, 28.9)	28.6 (25, 32.1)	3.5 (0.9, 6)	**0.009**
Light PA, min/day	286 (254, 319)	283 (254, 313)	−3 (−38, 32)	0.869	251 (214, 288)	281 (245, 316)	29 (0, 59)	0.053
MVPA, wear time %	1.7 (0.7, 2.6)	2 (1.3, 2.6)	0.3 (−0.6, 1.2)	0.538	1 (0.1, 1.9)	1.3 (0.4, 2.2)	0.3 (−0.2, 0.8)	0.268
MVPA, min/day	17.3 (7.7, 26.9)	18.7 (12.9, 24.5)	1.4 (−7.5, 10.4)	0.753	11.1 (1.9, 20.4)	11.9 (2.9, 21)	0.8 (−5.1, 6.7)	0.788
Steps/day	5556 (4103, 7008)	5787 (4773, 6800)	231 (−876, 1339)	0.678	4222 (3152, 5292)	5038 (3686, 6389)	816 (−173, 1804)	0.104
**Weekend**								
Light PA, wear time %	28.9 (24.7, 33)	28.9 (24.5, 33.2)	0 (−4.1, 4.2)	0.995	24.5 (19, 29.9)	31 (27.7, 34.3)	6.5 (1.7, 11.3)	**0.008**
Light PA, min/day	277 (240, 314)	275 (232, 318)	−2 (−44, 40)	0.931	235 (185, 284)	295 (265, 325)	61 (16, 106)	**0.009**
MVPA, wear time %	1 (0.4, 1.7)	1.3 (0.5, 2)	0.3 (−0.5, 1)	0.462	0.6 (0, 1.3)	0.7 (0.3, 1.1)	0 (−0.3, 0.4)	0.815
MVPA, min/day	9.9 (3.7, 16)	11.9 (4.8, 19)	1.3 (−3.3, 6)	0.520	6.7 (−0.3, 13.7)	6.5 (2.4, 10.6)	−0.2 (−4.4, 3.9)	0.911
Steps/day	4451 (3519, 5383)	4976 (3775, 6178)	525 (−430, 1480)	0.275	3484 (2404, 4564)	4460 (3551, 5369)	976 (−109, 2061)	0.077

Values are expressed as estimated marginal means (EMM), coefficient estimates (β), and 95% Wald confidence intervals (CI). ^a^ The models were analyzed using a generalized linear mixed model controlling for the daily accelerometer wearing time, except for the models of measures of wear time %. Bold values indicate significance at *p* < 0.05. Abbreviations: MVPA, moderate-vigorous physical activity; PA, physical activity.

**Table 3:** Patterns of objectively measured physical activity and sedentary behavior before and after COVID-19 vaccination in older adults with hypertension according to the housing type.

	Detached House				Apartment/Row House			
	June 2020	July 2021			June 2020	July 2021		
	EMM (95% CI)	EMM (95% CI)	β (95% CI) ^a^	*p*	EMM (95% CI)	EMM (95% CI)	β (95% CI) ^a^	*p*
**SEDENTARY BEHAVIOR**								
**Weekdays**								
Sedentary bouts ≥ 10 min, bouts/day	20.1 (18.1, 22.1)	19.7 (18.4, 20.9)	−0.4 (−2.6, 1.7)	0.692	21.3 (19.8, 22.7)	20.1 (18.6, 21.7)	−1.1 (−2.7, 0.4)	0.158
Sedentary bouts ≥ 30 min, bouts/day	3.9 (3.4, 4.5)	4.3 (3.5, 5.1)	0.4 (−0.5, 1.3)	0.435	5.1 (4.2, 6)	4.7 (4, 5.3)	−0.4 (−1.2, 0.3)	0.260
Sedentary bouts ≥ 10 min, min/day	441 (394, 489)	450 (408, 493)	9 (−41, 59)	0.721	502 (448, 556)	466 (417, 514)	−37 (−82, 9)	0.113
Sedentary bouts ≥ 30 min, min/day	184 (158, 210)	205 (165, 245)	21 (−19, 62)	0.299	241 (192, 290)	215 (180, 250)	−26 (−63, 11)	0.162
Length of sedentary bouts, min/day	6.8 (6.2, 7.5)	6.8 (6, 7.6)	0 (−0.8, 0.7)	0.940	7.8 (6.8, 8.7)	6.9 (6.3, 7.6)	−0.9 (−1.6, −0.1)	**0.020**
Breaks ≥ 1 min, breaks/day	102 (96, 109)	104 (95, 112)	1.1 (−6.5, 8.8)	0.770	98 (89, 106)	102 (96, 108)	4.4 (−1.4, 10.2)	0.133
Breaks ≥ 5 min, breaks/day	16.6 (13.2, 20)	17.1 (14.2, 19.9)	0.5 (−2.1, 3)	0.713	12.9 (9.7, 16.1)	16.3 (13.2, 19.4)	3.5 (0.7, 6.2)	**0.015**
Break rate ≥ 1 min, breaks/h	6.2 (5.8, 6.6)	6.3 (5.8, 6.8)	0.1 (−0.4, 0.6)	0.675	5.9 (5.4, 6.4)	6.2 (5.8, 6.5)	0.3 (−0.1, 0.6)	0.119
Break rate ≥ 5 min, breaks/h	1 (0.8, 1.2)	1 (0.9, 1.2)	0 (−0.1, 0.2)	0.602	0.8 (0.6, 1)	1 (0.8, 1.2)	0.2 (0.1, 0.4)	**0.009**
**Weekend**								
Sedentary bouts ≥ 10 min, bouts/day	20.3 (18.2, 22.4)	18.7 (16.6, 20.7)	−1.6 (−3.7, 0.4)	0.120	20.3 (18.2, 22.5)	18.1 (16.2, 19.9)	−2.2 (−4.3, −0.1)	**0.036**
Sedentary bouts ≥ 30 min, bouts/day	4.2 (3.1, 5.2)	4.9 (3.7, 6.1)	0.7 (−0.6, 2.1)	0.287	4.9 (3.6, 6.1)	3.4 (2.7, 4.1)	−1.4 (−2.6, −0.3)	**0.012**
Sedentary bouts ≥ 10 min, min/day	448 (398, 499)	439 (376, 502)	−9 (−60, 42)	0.725	486 (422, 551)	392 (346, 438)	−94 (−155, −33)	**0.003**
Sedentary bouts ≥ 30 min, min/day	189 (139, 239)	216 (161, 271)	27 (−24, 78)	0.294	235 (171, 300)	157 (123, 190)	−79 (−137, −21)	**0.008**
Length of sedentary bouts, min/day	7.3 (6.4, 8.2)	7.3 (6.2, 8.3)	0 (−0.8, 0.7)	0.942	7.9 (6.7, 9.1)	6.3 (5.6, 7)	−1.6 (−2.9, −0.3)	**0.014**
Breaks ≥ 1 min, breaks/day	93.8 (87.2, 100.3)	95.2 (88, 102.5)	1.5 (5.8, 8.5)	0.680	93.4 (84, 102.9)	106.7 (97.7, 115.7)	13.3 (3.1, 23.4)	**0.011**
Breaks ≥ 5 min, breaks/day	15.3 (12.4, 18.2)	15.8 (12.1, 19.6)	0.5 (−2.6, 3.7)	0.729	10.9 (7.6, 14.3)	17.4 (14.8, 20.1)	6.5 (3.1, 9.8)	**<0.001**
Break rate ≥ 1 min, breaks/h	5.9 (5.5, 6.4)	6 (5.6, 6.5)	0.1 (−0.4, 0.5)	0.767	5.9 (5.3, 6.5)	6.7 (6.2, 7.2)	0.8 (0.2, 1.4)	**0.015**
Break rate ≥ 5 min, breaks/h	1 (0.8, 1.1)	1 (0.8, 1.2)	0 (−0.2, 0.2)	0.694	0.7 (0.5, 0.9)	1.1 (0.9, 1.3)	0.4 (0.2, 0.6)	**<0.001**
**PHYSICAL ACTIVITY**								
**Weekdays**								
Light PA in bouts ≥ 10 min, bouts/day	3.7 (2.8, 4.6)	3.1 (2.1, 4.2)	−0.6 (−1.5, 0.4)	0.240	3 (1.8, 4.2)	3.4 (1.9, 4.9)	0.4 (−0.8, 1.6)	0.514
Light PA in bouts ≥ 10 min, min/day	54.6 (40.6, 68.5)	46.2 (27.6, 64.8)	−8.4 (−26.1, 9.4)	0.350	41.8 (24.3, 59.4)	47.1 (24, 70.3)	5.3 (−12.4, 22.9)	0.552
MVPA in bouts ≥ 10 min, bouts/day	0.2 (0.1, 0.4)	0.2 (0.1, 0.4)	0.0 (−0.3, 0.2)	0.819	0.2 (0.0, 0.5)	0.2 (0.0, 0.4)	0.0 (−0.2, 0.2)	0.851
MVPA in bouts ≥ 10 min, min/day	4.5 (1.3, 7.7)	4.5 (0.6, 8.4)	0 (−5.1, 5)	0.990	7.4 (−1.3, 16.1)	3.7 (−1.4, 8.8)	−3.7 (−9.1, 1.8)	0.185
**Weekend**								
Light PA in bouts ≥ 10 min, bouts/day	4.5 (3, 6)	3.8 (2.5, 5.1)	0.7 (−2, 0.6)	0.300	2.9 (1, 4.7)	3.2 (2, 4.4)	0.3 (−1.2, 1.9)	0.667
Light PA in bouts ≥ 10 min, min/day	67.2 (43.2, 91.2)	59.2 (37.1, 81.3)	−8 (−29.6, 13.6)	0.462	44.4 (9.7, 79.2)	44.1 (27.4, 60.9)	−0.3 (−28.8, 28.2)	0.983
MVPA in bouts ≥ 10 min, bouts/day	0.1 (−0.1, 0.4)	0.2 (−0.1, 0.5)	0.1 (−0.2, 0.3)	0.447	0.1 (0, 0.3)	0 (0, 0.1)	−0.1 (−0.3, 0)	0.120
MVPA in bouts ≥ 10 min, min/day	1.4 (−1.2, 4)	4.3 (−2.1, 10.7)	2.9 (−3.4, 9.1)	0.359	5 (−1.9, 11.8)	1.3 (−1.5, 4)	−3.7 (−10.4, 3)	0.274

Values are expressed as estimated marginal means (EMM), coefficient estimates (β), and 95% Wald confidence intervals (CI). ^a^ The models were analyzed using a generalized linear mixed model controlling for the daily accelerometer wearing time, except for the length of sedentary bouts and the break rate. Bold values indicate significance at *p* < 0.05. Abbreviations: MVPA, moderate-vigorous physical activity; PA, physical activity.

## Data Availability

https://osf.io/wv5ye/?view_only=a2a5b0cd6a69433b8a7e57d11539b4ec (accessed on 29 July 2022).

## Supplementary Materials

The following supporting information can be downloaded at: https://www.mdpi.com/article/10.3390/ijerph191912532/s1, Table S1—Moderating effect of housing type on objectively measured changes in the volume of physical activity and sedentary behavior after COVID-19 vaccination in older adults with hypertension (*n* = 32); Table S2—Moderating effect of housing type on objectively measured changes in the pattern of physical activity and sedentary behavior after COVID-19 vaccination in older adults with hypertension (*n* = 32); Table S3—Associations of increased life-space mobility with objectively measured changes in the volume of physical activity and sedentary behavior after COVID-19 vaccination in older adults with hypertension residing in apartment/row housing (*n* = 15); Table S4—Associations of increased life-space mobility with objectively measured changes in the physical activity and sedentary behavior pattern after COVID-19 vaccination in older adults with hypertension residing in apartment/row housing (*n* = 15).

## References

[1] World Health Organization (2022). Weekly Epidemiological Update on COVID-19—31 August 2022.

[2] Aquino E.M.L., Silveira I.H., Pescarini J.M., Aquino R., De Souza-Filho J.A., Rocha A.D.S., Ferreira A., Victor A., Teixeira C., Machado D.B. (2020). Social distancing measures to control the COVID-19 pandemic: Potential impacts and challenges in Brazil. Ciência Saúde Coletiva.

[3] Yang J., Zheng Y., Gou X., Pu K., Chen Z., Guo Q., Ji R., Wang H., Wang Y., Zhou Y. (2020). Prevalence of comorbidities and its effects in patients infected with SARS-CoV-2: A systematic review and meta-analysis. Int. J. Infect. Dis..

[4] Espinosa O.A., Zanetti A.D.S., Antunes E.F., Longhi F.G., De Matos T.A., Battaglini P.F. (2020). Prevalence of comorbidities in patients and mortality cases affected by SARS-CoV2: A systematic review and meta-analysis. Rev. Inst. Med. Trop. São Paulo.

[5] Perracini M.R., de Amorim J.S.C., Lima C.A., da Silva A., Trombini-Souza F., Pereira D.S., Pelicioni P.H.S., Duim E., Batista P.P., dos Santos R.B. (2021). Impact of COVID-19 Pandemic on Life-Space Mobility of Older Adults Living in Brazil: REMOBILIZE Study. Front. Public Health.

[6] Rantanen T., Eronen J., Kauppinen M., Kokko K., Sanaslahti S., Kajan N., Portegijs E. (2020). Life-Space Mobility and Active Aging as Factors Underlying Quality of Life Among Older People Before and During COVID-19 Lockdown in Finland—A Longitudinal Study. J. Gerontol. Ser. A.

[7] Saraiva M.D., Apolinario D., Avelino-Silva T.J., Tavares C.D.A.M., Gattás-Vernaglia I.F., Fernandes C.M., Rabelo L.M., Yamaguti S.T.F., Karnakis T., Kalil-Filho R. (2020). The Impact of Frailty on the Relationship between Life-Space Mobility and Quality of Life in Older Adults during the COVID-19 Pandemic. J. Nutr. Health Aging.

[8] Taylor J.K., Buchan I.E., van der Veer S.N. (2018). Assessing life-space mobility for a more holistic view on wellbeing in geriatric research and clinical practice. Aging Clin. Exp. Res..

[9] Browne R.A.V., Macêdo G.A.D., Cabral L.L.P., Oliveira G.T.A., Vivas A., Fontes E.B., Elsangedy H.M., Costa E.C. (2020). Initial impact of the COVID-19 pandemic on physical activity and sedentary behavior in hypertensive older adults: An accelerometer-based analysis. Exp. Gerontol..

[10] Oliveira M.R., Sudati I.P., Konzen V.D.M., de Campos A.C., Wibelinger L.M., Correa C., Miguel F.M., Silva R.N., Borghi-Silva A. (2021). Covid-19 and the impact on the physical activity level of elderly people: A systematic review. Exp. Gerontol..

[11] Pérez-Gisbert L., Torres-Sánchez I., Ortiz-Rubio A., Calvache-Mateo A., López-López L., Cabrera-Martos I., Valenza M.C. (2021). Effects of the COVID-19 Pandemic on Physical Activity in Chronic Diseases: A Systematic Review and Meta-Analysis. Int. J. Environ. Res. Public Health.

[12] Runacres A., Mackintosh K.A., Knight R.L., Sheeran L., Thatcher R., Shelley J., McNarry M.A. (2021). Impact of the COVID-19 Pandemic on Sedentary Time and Behaviour in Children and Adults: A Systematic Review and Meta-Analysis. Int. J. Environ. Res. Public Health.

[13] Browne R.A.V., Cabral L.L.P., Freire Y.A., Macêdo G.A.D., Oliveira G.T.A., Vivas A., Elsangedy H.M., Fontes E.B., Costa E.C. (2021). Housing type is associated with objectively measured changes in movement behavior during the COVID-19 pandemic in older adults with hypertension: An exploratory study. Arch. Gerontol. Geriatr..

[14] Fan Y.-J., Chan K.-H., Hung I.F.-N. (2021). Safety and Efficacy of COVID-19 Vaccines: A Systematic Review and Meta-Analysis of Different Vaccines at Phase 3. Vaccines.

[15] Pormohammad A., Zarei M., Ghorbani S., Mohammadi M., Razizadeh M., Turner D., Turner R. (2021). Efficacy and Safety of COVID-19 Vaccines: A Systematic Review and Meta-Analysis of Randomized Clinical Trials. Vaccines.

[16] Xing K., Tu X.-Y., Liu M., Liang Z.-W., Chen J.-N., Li J.-J., Jiang L.-G., Xing F.-Q., Jiang Y. (2021). Efficacy and safety of COVID-19 vaccines: A systematic review. Chin. J. Contemp. Pediatrics.

[17] Voysey M., Clemens S.A.C., Madhi S.A., Weckx L.Y., Folegatti P.M., Aley P.K., Angus B., Baillie V.L., Barnabas S.L., Bhorat Q.E. (2021). Safety and efficacy of the ChAdOx1 nCoV-19 vaccine (AZD1222) against SARS-CoV-2: An interim analysis of four randomised controlled trials in Brazil, South Africa, and the UK. Lancet.

[18] González S., Olszevicki S., Salazar M., Calabria A., Regairaz L., Marín L., Campos P., Varela T., Martínez V.V.G., Ceriani L. (2021). Effectiveness of the first component of Gam-COVID-Vac (Sputnik V) on reduction of SARS-CoV-2 confirmed infections, hospitalisations and mortality in patients aged 60-79: A retrospective cohort study in Argentina. eClinicalMedicine.

[19] Haas E.J., Angulo F.J., McLaughlin J.M., Anis E., Singer S.R., Khan F., Brooks N., Smaja M., Mircus G., Pan K. (2021). Impact and effectiveness of mRNA BNT162b2 vaccine against SARS-CoV-2 infections and COVID-19 cases, hospitalisations, and deaths following a nationwide vaccination campaign in Israel: An observational study using national surveillance data. Lancet.

[20] Hitchings M.D.T., Ranzani O.T., Torres M.S.S., de Oliveira S.B., Almiron M., Said R., Borg R., Schulz W.L., de Oliveira R.D., da Silva P.V. (2021). Effectiveness of CoronaVac among healthcare workers in the setting of high SARS-CoV-2 Gamma variant transmission in Manaus, Brazil: A test-negative case-control study. Lancet Reg. Health Am..

[21] Ranzani O.T., Hitchings M.D.T., Dorion M., D’Agostini T.L., de Paula R.C., de Paula O.F.P., Villela E.F.D.M., Torres M.S.S., de Oliveira S.B., Schulz W. (2021). Effectiveness of the CoronaVac vaccine in older adults during a gamma variant associated epidemic of covid-19 in Brazil: Test negative case-control study. BMJ.

[22] Victora C.G., Castro M.C., Gurzenda S., Medeiros A.C., França G.V., Barros A.J. (2021). Estimating the early impact of vaccination against COVID-19 on deaths among elderly people in Brazil: Analyses of routinely-collected data on vaccine coverage and mortality. eClinicalMedicine.

[23] Björk J., Inghammar M., Moghaddassi M., Rasmussen M., Malmqvist U., Kahn F. (2021). High level of protection against COVID-19 after two doses of BNT162b2 vaccine in the working age population—First results from a cohort study in Southern Sweden. Infect. Dis..

[24] Hall V.J., Foulkes S., Saei A., Andrews N., Oguti B., Charlett A., Wellington E., Stowe J., Gillson N., Atti A. (2021). COVID-19 vaccine coverage in health-care workers in England and effectiveness of BNT162b2 mRNA vaccine against infection (SIREN): A prospective, multicentre, cohort study. Lancet.

[25] Dagan N., Barda N., Kepten E., Miron O., Perchik S., Katz M.A., Hernán M.A., Lipsitch M., Reis B., Balicer R.D. (2021). BNT162b2 mRNA Covid-19 Vaccine in a Nationwide Mass Vaccination Setting. N. Engl. J. Med..

[26] Bernal J.L., Andrews N., Gower C., Robertson C., Stowe J., Tessier E., Simmons R., Cottrell S., Roberts R., O’Doherty M. (2021). Effectiveness of the Pfizer-BioNTech and Oxford-AstraZeneca vaccines on covid-19 related symptoms, hospital admissions, and mortality in older adults in England: Test negative case-control study. BMJ.

[27] Centers for Disease Control and Prevention (CDC) (2021). Interim Public Health Recommendations for Fully Vaccinated People. https://www.cdc.gov/coronavirus/2019-ncov/vaccines/fully-vaccinated-guidance.html.

[28] Brazil Ministry of Health (2021). Painel Coronavírus. https://covid.saude.gov.br.

[29] Brazil Ministry of Health (2020). Secretariat of Health Surveillance. Special Epidemiological Bulletin 18. Coronavirus Disease—COVID-19. https://www.gov.br/saude/pt-br/centrais-de-conteudo/publicacoes/boletins/epidemiologicos/covid-19/2020/boletim-epidemiologico-no-18-boletim-coe-coronavirus.pdf/view.

[30] State Department of Public Health of Rio Grande do Norte (2020). Epidemiological Bulletin 76—Coronavirus Disease (COVID-19). https://portalcovid19.saude.rn.gov.br/wp-content/uploads/2020/04/76_boletim_covid-19-1.pdf.

[31] Laboratory of Technological Innovation in Health (2021). Regulatory of Intensive Care Unit Beds of Rio Grande do Norte, Brazil. In: Federal University of Rio Grande do Norte. https://regulacao.saude.rn.gov.br/sala-situacao/sala_publica/.

[32] Brazil Ministry of Health (2021). Secretariat of Health Surveillance. Special Epidemiological Bulletin 71. Coronavirus Disease—COVID-19. https://www.gov.br/saude/pt-br/centrais-de-conteudo/publicacoes/boletins/epidemiologicos/covid-19/2021/boletim_epidemiologico_covid_71.pdf/view.

[33] State Department of Public Health of Rio Grande do Norte (2021). Coronavirus Epidemiological Report (COVID-19). Special Edition—Epidemiological Week 27. https://portalcovid19.saude.rn.gov.br/wp-content/uploads/2020/04/boletim-especial-SE-27.pdf.

[34] Svensson A.C., Forsberg J.S., Seblova D., Lager A. (2016). Residential area and physical activity: A multi-level study of 68,000 adults in Stockholm County. Scand. J. Public Health.

[35] McKee G., Kearney P., Kenny R.A. (2015). The factors associated with self-reported physical activity in older adults living in the community. Age Ageing.

[36] Saidj M., Jørgensen T., Jacobsen R.K., Linneberg A., Aadahl M. (2015). The influence of housing characteristics on leisure-time sitting. A prospective cohort study in Danish adults. Prev. Med..

[37] Pettigrew S., Rai R., Jongenelis M.I., Jackson B., Beck B., Newton R.U. (2019). The Potential Importance of Housing Type for Older People’s Physical Activity Levels. J. Appl. Gerontol..

[38] Choi L., Liu Z., Matthews C.E., Buchowski M.S. (2011). Validation of Accelerometer Wear and Nonwear Time Classification Algorithm. Med. Sci. Sports Exerc..

[39] Trost S.G., Mciver K.L., Pate R.R. (2005). Conducting Accelerometer-Based Activity Assessments in Field-Based Research. Med. Sci. Sports Exerc..

[40] Migueles J.H., Cadenas-Sanchez C., Ekelund U., Delisle Nyström C., Mora-Gonzalez J., Löf M., Labayen I., Ruiz J.R., Ortega F.B. (2017). Accelerometer Data Collection and Processing Criteria to Assess Physical Activity and Other Outcomes: A Systematic Review and Practical Considerations. Sports Med..

[41] Matthews C.E., Chen K.Y., Freedson P.S., Buchowski M.S., Beech B.M., Pate R.R., Troiano R.P. (2008). Amount of Time Spent in Sedentary Behaviors in the United States, 2003–2004. Am. J. Epidemiol..

[42] Freedson P.S., Melanson E., Sirard J. (1998). Calibration of the Computer Science and Applications, Inc. accelerometer. Med. Sci. Sports Exerc..

[43] Boerema S.T., Van Velsen L., Vollenbroek M., Hermens H.J. (2020). Pattern measures of sedentary behaviour in adults: A literature review. Digit. Health.

[44] Simões M.D.S.M., Garcia I.F., Costa L.D.C., Lunardi A.C. (2018). Life-Space Assessment questionnaire: Novel measurement properties for Brazilian community-dwelling older adults. Geriatr. Gerontol. Int..

[45] Peel C., Baker P.S., Roth D.L., Brown C.J., Brodner E.V., Allman R.M. (2005). Assessing mobility in older adults: The UAB Study of Aging Life-Space Assessment. Phys. Ther..

[46] Sawyer P., Allman R.M., Fry P.S., Keyes C.L.M. (2010). Resilience in mobility in the context of chronic disease and aging. New Frontiers in Resilient Aging.

[47] Baker P.S., Bodner E.V., Allman R.M. (2003). Measuring Life-Space Mobility in Community-Dwelling Older Adults. J. Am. Geriatr. Soc..

[48] Kennedy R.E., Sawyer P., Mph C.P.W., Lo A.X., Ritchie C.S., Roth D.L., Allman R.M., Brown C.J. (2017). Life-Space Mobility Change Predicts 6-Month Mortality. J. Am. Geriatr. Soc..

[49] Kammerlind A.-S.C., Fristedt S., Bravell M.E., Fransson E. (2014). Test–retest reliability of the Swedish version of the Life-Space Assessment Questionnaire among community-dwelling older adults. Clin. Rehabil..

[50] Babicki M., Malchrzak W., Hans-Wytrychowska A., Mastalerz-Migas A. (2021). Impact of Vaccination on the Sense of Security, the Anxiety of COVID-19 and Quality of Life among Polish. A Nationwide Online Survey in Poland. Vaccines.

[51] Boto-García D., Pino J.F.B. (2022). Propelled: Evidence on the impact of vaccination against COVID-19 on travel propensity. Curr. Issues Tour..

[52] Portegijs E., Rantakokko M., Viljanen A., Sipila S., Rantanen T. (2016). Is frailty associated with life-space mobility and perceived autonomy in participation outdoors? A longitudinal study. Age Ageing.

[53] Brown C.J. (2009). Trajectories of Life-Space Mobility After Hospitalization. Ann. Intern. Med..

[54] Galmes-Panades A.M., Varela-Mato V., Konieczna J., Wärnberg J., Martínez-González M.Á., Salas-Salvadó J., Corella D., Schröder H., Vioque J., Alonso-Gómez Á.M. (2019). Isotemporal substitution of inactive time with physical activity and time in bed: Cross-sectional associations with cardiometabolic health in the PREDIMED-Plus study. Int. J. Behav. Nutr. Phys. Act..

[55] Del Pozo-Cruz J., García-Hermoso A., Alfonso-Rosa R.M., Alvarez-Barbosa F., Owen N., Chastin S., del Pozo-Cruz B. (2018). Replacing Sedentary Time: Meta-analysis of Objective-Assessment Studies. Am. J. Prev. Med..

[56] Dempsey P.C., Owen N., Yates T., Kingwell B.A., Dunstan D.W. (2016). Sitting Less and Moving More: Improved Glycaemic Control for Type 2 Diabetes Prevention and Management. Curr. Diabetes Rep..

[57] Dempsey P.C., Larsen R.N., Dunstan D.W., Owen N., Kingwell B.A. (2018). Sitting Less and Moving More: Implications for hypertension. Hypertension.

