# Supplementary material for: Life-Space Mobility and Objectively Measured Movement Behavior in Older Adults with Hypertension after Receiving COVID-19 Vaccination
Source: Int J Environ Res Public Health. 2022 Oct 1;19(19):12532. doi: 10.3390/ijerph191912532 (PMC9566032; doi:10.3390/ijerph191912532)
Supplement: Supplementary file 1 [file ijerph-19-12532-s001.zip › Table S1.pdf]

Table S1. Moderating effect of housing type on objectively measured changes in the volume of physical activity and sedentary behavior after COVID-19 vaccination in older adults with hypertension (n = 32).

	β	SE	95% CI	p [a]
**SEDENTARY BEHAVIOR**				
**Weekdays**				
Sedentary, wear time %	–3.7	2.2	–8.1, 0.7	0.101
Sedentary, min/day	–31.5	22.0	–75.6, 12.6	0.158
**Weekend**				
Sedentary, wear time %	–6.3	3.2	–12.7, 0.1	**0.055**
Sedentary, min/day	–60.0	30.7	–121.4, 1.3	**0.055**
**PHYSICAL ACTIVITY**				
**Weekdays**				
Light PA, wear time %	3.7	2.1	–0.6, 7.9	**0.090**
Light PA, min/day	32.1	21.5	–10.9, 75.2	0.141
MVPA, wear time %	0.0	0.5	–1.0, 1.0	0.993
MVPA, min/day	–0.6	4.8	–10.2, 9.0	0.897
Steps/day	585	655	–726, 1895	0.376
**Weekend**				
Light PA, wear time %	6.5	3.2	0.2, 12.9	**0.044**
Light PA, min/day	62.4	30.5	1.3, 123.5	**0.045**
MVPA, wear time %	–0.2	0.4	–1.0, 0.6	0.584
MVPA, min/day	–2.3	3.6	–9.5, 4.9	0.525
Steps/day	451	723	–996, 1898	0.535

Values are expressed as coefficient estimates (β), standard error (SE) and 95% Wald confidence intervals (CI) of the housing type by time period interaction (i.e. change in apartment/row house vs. change in detached house – reference group). [a] The models were analyzed using a generalized linear mixed model controlling for the daily accelerometer wearing time, except for the models of measures of wear time %. Bold values indicate significance at $p < 0.10$. Abbreviations: MVPA, moderate-vigorous physical activity; PA, physical activity.
