# Supplementary material for: Life-Space Mobility and Objectively Measured Movement Behavior in Older Adults with Hypertension after Receiving COVID-19 Vaccination
Source: Int J Environ Res Public Health. 2022 Oct 1;19(19):12532. doi: 10.3390/ijerph191912532 (PMC9566032; doi:10.3390/ijerph191912532)
Supplement: Supplementary file 1 [file ijerph-19-12532-s001.zip › Table S2.pdf]

Table S2. Moderating effect of housing type on objectively measured changes in the pattern of physical activity and sedentary behavior after COVID-19 vaccination in older adults with hypertension (n = 32).

	β	SE	95% CI	p [a]
**SEDENTARY BEHAVIOR**				
**Weekdays**				
Sedentary bouts ≥ 10 min, bouts/day	–0.7	1.2	–3.0, 1.6	0.555
Sedentary bouts ≥ 30 min, bouts/day	–0.8	0.6	–1.9, 0.3	0.162
Sedentary bouts ≥ 10 min, min/day	–45.6	31.3	–108.3, 17.0	0.150
Sedentary bouts ≥ 30 min, min/day	–47.3	26.0	–99.4, 4.9	**0.075**
Length of sedentary bouts, min/day	–0.8	0.5	–1.8, 0.1	**0.091**
Breaks ≥ 1 min, breaks/day	3.3	4.6	–5.9, 12.5	0.478
Breaks ≥ 5 min, breaks/day	3.0	1.6	–0.2, 6.2	**0.066**
Break rate ≥ 1 min, breaks/h	0.2	0.3	–0.4, 0.7	0.495
Break rate ≥ 5 min, breaks/h	0.2	0.1	0.0, 0.4	**0.052**
**Weekend**				
Sedentary bouts ≥ 10 min, bouts/day	–0.6	1.4	–3.5, 2.3	0.672
Sedentary bouts ≥ 30 min, bouts/day	–2.2	0.9	–3.9, –0.4	**0.015**
Sedentary bouts ≥ 10 min, min/day	–85.2	38.9	–163.1, –7.3	**0.033**
Sedentary bouts ≥ 30 min, min/day	–106	37	–181, –31	**0.006**
Length of sedentary bouts, min/day	–1.6	0.7	–3.0, –0.2	**0.029**
Breaks ≥ 1 min, breaks/day	11.8	6.0	–0.2, 23.8	**0.053**
Breaks ≥ 5 min, breaks/day	5.9	2.3	1.2, 10.6	**0.014**
Break rate ≥ 1 min, breaks/h	0.7	0.4	0.0, 1.5	**0.052**
Break rate ≥ 5 min, breaks/h	0.4	0.1	0.1, 0.7	**0.014**
**PHYSICAL ACTIVITY**				
**Weekdays**				
Light PA in bouts ≥ 10 min, bouts/day	1.0	0.7	–0.5, 2.4	0.194
Light PA in bouts ≥ 10 min, min/day	13.6	12.5	–11.4, 38.7	0.281
MVPA in bouts ≥ 10 min, bouts/day	0.0	0.1	–0.3, 0.3	0.943
MVPA in bouts ≥ 10 min, min/day	–3.6	3.1	–9.8, 2.6	0.249
**Weekend**				
Light PA in bouts ≥ 10 min, bouts/day	1.0	1.0	–1.0, 3.0	0.326
Light PA in bouts ≥ 10 min, min/day	7.7	18.4	–29.1, 44.5	0.677
MVPA in bouts ≥ 10 min, bouts/day	–0.2	0.1	–0.5, 0.1	0.132
MVPA in bouts ≥ 10 min, min/day	–6.6	4.2	–15.0, 1.9	0.125

Values are expressed as coefficient estimates (β), standard error (SE) and 95% Wald confidence intervals (CI) of the housing type by time period interaction (i.e. change in apartment/row house vs. change in detached house – reference group). [a] The models were analyzed using a generalized linear mixed model controlling for the daily accelerometer wearing time, except for the length of sedentary bouts and the break rate. Bold values indicate significance at $p < 0.10$. Abbreviations: MVPA, moderate-vigorous physical activity; PA, physical activity.
