# Supplementary material for: Life-Space Mobility and Objectively Measured Movement Behavior in Older Adults with Hypertension after Receiving COVID-19 Vaccination
Source: Int J Environ Res Public Health. 2022 Oct 1;19(19):12532. doi: 10.3390/ijerph191912532 (PMC9566032; doi:10.3390/ijerph191912532)
Supplement: Supplementary file 1 [file ijerph-19-12532-s001.zip › Table S3.pdf]

Table S3. Associations of increased life-space mobility with objectively measured changes in the volume of physical activity and sedentary behavior after COVID-19 vaccination in older adults with hypertension residing in apartment/row housing (*n* = 15).

	β	SE	95% CI	*p* [a]
**SEDENTARY BEHAVIOR**				
**Weekdays**				
Sedentary, wear time %	–3.1	2.7	–8.5, 2.4	0.261
Sedentary, min/day	–29.6	28.7	–88.7, 29.5	0.312
**Weekend**				
Sedentary, wear time %	–7.8	4.0	–16.1, 0.5	**0.065**
Sedentary, min/day	–68.5	39.6	–150.0, 13.1	**0.096**
**PHYSICAL ACTIVITY**				
**Weekdays**				
Light PA, wear time %	2.2	2.6	–3.0, 7.5	0.390
Light PA, min/day	19.9	27.6	–36.9, 76.7	0.477
MVPA, wear time %	0.8	0.4	–1.0, 1.7	**0.068**
MVPA, min/day	9.7	4.9	–0.5, 19.9	**0.060**
Steps/day	1818	730	314, 3321	**0.020**
**Weekend**				
Light PA, wear time %	8.3	4.1	–0.2, 16.7	**0.054**
Light PA, min/day	73.2	40.5	–10.3, 156.6	**0.083**
MVPA, wear time %	–0.5	0.4	–1.3, 0.2	0.163
MVPA, min/day	–4.5	3.2	–11.1, 2.1	0.175
Steps/day	966	1017	–1128, 3060	0.351

Values are expressed as coefficient estimates (β), standard error (SE) and 95% Wald confidence intervals (CI) of the increased life-space mobility by time period interaction (i.e. change in increased life-space group vs. change in change in non-increased life-space group – reference group). [a] The models were analyzed using a generalized linear mixed model controlling for the daily accelerometer wearing time, except for the models of measures of wear time %. Bold values indicate significance at $p < 0.10$. Abbreviations: MVPA, moderate-vigorous physical activity; PA, physical activity.
