# Supplementary material for: Life-Space Mobility and Objectively Measured Movement Behavior in Older Adults with Hypertension after Receiving COVID-19 Vaccination
Source: Int J Environ Res Public Health. 2022 Oct 1;19(19):12532. doi: 10.3390/ijerph191912532 (PMC9566032; doi:10.3390/ijerph191912532)
Supplement: Supplementary file 1 [file ijerph-19-12532-s001.zip › Table S4.pdf]

Table S4. Associations of increased life-space mobility with objectively measured changes in the physical activity and sedentary behavior pattern after COVID-19 vaccination in older adults with hypertension residing in apartment/row housing (*n* = 15).

	β	SE	95% CI	*p* [a]
**SEDENTARY BEHAVIOR**				
**Weekdays**				
Sedentary bouts ≥ 10 min, bouts/day	–1.6	1.3	–4.3, 1.2	0.251
Sedentary bouts ≥ 30 min, bouts/day	–0.9	0.9	–2.7, 0.8	0.285
Sedentary bouts ≥ 10 min, min/day	–44.4	44.6	–136.3, 47.6	0.330
Sedentary bouts ≥ 30 min, min/day	–36.7	39.5	–117.9, 44.6	0.362
Length of sedentary bouts, min/day	–0.3	0.6	–1.6, 0.1	0.662
Breaks ≥ 1 min, breaks/day	–1.7	5.2	–12.4, 9.0	0.745
Breaks ≥ 5 min, breaks/day	3.5	2.3	–1.2, 8.2	0.134
Break rate ≥ 1 min, breaks/h	–0.2	0.3	–0.8, 0.4	0.587
Break rate ≥ 5 min, breaks/h	0.2	0.1	–0.1, 0.5	0.125
**Weekend**				
Sedentary bouts ≥ 10 min, bouts/day	–4.2	1.7	–7.8, –0.7	**0.022**
Sedentary bouts ≥ 30 min, bouts/day	–1.4	1.1	–3.6, 0.9	0.215
Sedentary bouts ≥ 10 min, min/day	–76.4	58.0	–195.9, 43.1	0.200
Sedentary bouts ≥ 30 min, min/day	–38.1	56.9	–155.2, 79.0	0.509
Length of sedentary bouts, min/day	–0.7	1.1	–3.0, 1.6	0.557
Breaks ≥ 1 min, breaks/day	–0.8	10.2	–21.8, 20.3	0.942
Breaks ≥ 5 min, breaks/day	8.2	2.6	2.9, 13.5	**0.004**
Break rate ≥ 1 min, breaks/h	–0.1	0.6	–1.2, 1.1	0.890
Break rate ≥ 5 min, breaks/h	0.5	0.2	0.2, 0.9	**0.004**
**PHYSICAL ACTIVITY**				
**Weekdays**				
Light PA in bouts ≥ 10 min, bouts/day	1.1	1.2	–1.3, 3.5	0.337
Light PA in bouts ≥ 10 min, min/day	11.1	16.8	–23.6, 45.8	0.516
MVPA in bouts ≥ 10 min, bouts/day	0.3	0.2	–0.1, 0.7	0.109
MVPA in bouts ≥ 10 min, min/day	3.8	4.2	–4.8, 12.4	0.367
**Weekend**				
Light PA in bouts ≥ 10 min, bouts/day	3.0	1.3	0.2, 5.7	**0.038**
Light PA in bouts ≥ 10 min, min/day	52.0	24.8	1.0, 103.0	**0.046**
MVPA in bouts ≥ 10 min, bouts/day	–0.1	0.1	–0.3, 0.2	0.674
MVPA in bouts ≥ 10 min, min/day	–5.9	5.7	–17.8, 5.9	0.311

Values are expressed as coefficient estimates (β), standard error (SE) and 95% Wald confidence intervals (CI) of the increased life-space mobility by time period interaction (i.e. change in increased life-space group vs. change in non-increased life-space group – reference group). [a] The models were analyzed using a generalized linear mixed model controlling for the daily accelerometer wearing time, except for the length of sedentary bouts the and break rate. Bold values indicate significance at $p < 0.10$. Abbreviations: MVPA, moderate-vigorous physical activity; PA, physical activity.
